# Association between surrogate indicators of insulin resistance and risk of type 2 diabetes combined with hypertension among Chinese adults: two independent cohort studies

**DOI:** 10.1186/s12986-022-00720-1

**Published:** 2022-12-29

**Authors:** Jing Dong, Yu-Hong Liu, Ya-Ke Lu, Li-Kun Hu, Ning Chen, Lin-Lin Ma, Xi Chu, Yu-Xiang Yan

**Affiliations:** 1 Health Management Center, Xuanwu Hospital, Capital Medical University, No.45 Changchun Street, Xicheng District, Beijing, People’s Republic of China; 2 Department of Epidemiology and Biostatistics, School of Public Health, Capital Medical University, No.10 Xitoutiao, You’anmenWai, Fengtai District, 100069 Beijing, People’s Republic of China; 3 Municipal Key Laboratory of Clinical Epidemiology, Beijing, People’s Republic of China

**Keywords:** Hypertension, Insulin resistance, Type 2 diabetes, Obesity, Triglyceride-glucose index, Mets-IR

## Abstract

**Background:**

Type 2 diabetes (T2D) combined with hypertension has a higher risk of developing cardiovascular diseases. This study aimed to investigate the relationships between the surrogate indicators of insulin resistance (TyG, TG/HDL, and Mets-IR) with the risk of T2D combined with hypertension.

**Methods:**

This study is based on a functional community cohort from Beijing and the China Health and Retirement Longitudinal Study, comprising 4234 and 4658 participants respectively. Cox proportional hazards models and restricted cubic spline regression were performed to assess the link between TyG, TG/HDL, and Mets-IR with T2D combined with hypertension. The cross-lagged panel analysis and the mediation analysis were used to examine the temporal relationship between insulin resistance and obesity and their temporal relationship with follow-up T2D combined with hypertension.

**Results:**

In multivariable-adjusted models, higher TyG was associated with a higher risk of developing T2D combined with hypertension, the hazard ratios (95% confidence interval) were 3.46 (2.43–4.93) and 2.02 (1.67–2.44), in two cohorts respectively. A similar positive association was shown for Mets-IR, the hazard ratios (95% confidence interval) were 1.04 (1.03–1.06) and 1.05 (1.03–1.07), in two cohorts respectively. However, the association between TG/HDL with T2D combined with hypertension was different in two cohorts. The restricted cubic spline regression showed a linear association between TyG and T2D combined with hypertension (*P*-nonlinear > 0.05). The cross-lagged path coefficient from baseline BMI to follow-up TyG index was significantly greater than the path coefficient from baseline TyG to follow-up BMI. TyG partially mediated the effect of BMI on the risk of T2D combined with hypertension and the percentage of mediated association was estimated at 41.58% and 48.41% in two cohorts, respectively.

**Conclusion:**

These findings indicated positive associations between TyG and Mets-IR with the risk of T2D combined with hypertension in two cohorts. In addition, BMI change may precede TyG index change, and the TyG index plays a mediating role in BMI induced T2D combined with hypertension.

**Supplementary Information:**

The online version contains supplementary material available at 10.1186/s12986-022-00720-1.

## Introduction

Type 2 diabetes (T2D) and hypertension, as important risk factors for cardiovascular diseases, pose a tremendous medical and economic burden to the world [1]. We cannot ignore the fact that T2D and hypertension frequently occur together, not only are persons with T2D more prone to develop hypertension than the general population, but people with hypertension also have a higher risk of T2D [2, 3]. There is a huge diabetic and hypertensive population in China. Evidence from a multi-center study revealed that 45.3% of people had T2D combined with hypertension [4]. The coexistence of these two diseases significantly increases the risk of cardiovascular diseases. Hence, it is essential to identify those who are at a higher risk of developing T2D combined with hypertension to reduce the public health burden.

One important pathophysiological mechanism for the emergence of T2D is insulin resistance (IR) [5]. It will present a long time before diagnosis, and may go on to develop pre-diabetes or T2D if timely intervention and effective lifestyle changes are not made [6]. In addition, previous studies have highlighted the positive association of IR with blood pressure elevation [7, 8]. Participants with hypertension are often combined with abnormalities in glucolipid metabolism, and IR plays a significant role in this biological process [9]. With the improvement in living standards, the incidence of obesity and dyslipidemia has increased significantly [10, 11], which are major factors that contribute to IR [12, 13].

The euglycemic insulin clamp technique (EICT) is recognized as the gold standard of IR [14]. However, EICT is limited in clinical use due to its low practicality and expensive testing costs. Recently, the triglyceride and glucose index (TyG) [15], triglyceride to high-density lipoprotein ratio (TG/HDL-C) [16], and metabolic score for IR (Mets-IR) [17] have been proposed as the surrogate indicators of IR. TyG is fasting blood glucose and triglyceride synthesis index, TG/HDL is the ratio of fasting triglyceride to fasting high-density lipoprotein cholesterol, and Mets-IR is a novel indicator calculated by fasting glucose, fasting triglycerides, body mass index (BMI), and high-density lipoprotein cholesterol. These novel indexes are calculated from conventional biochemical test indexes with higher practicality.

Several studies have examined the association of the surrogate indicators of IR with T2D and hypertension in different populations [18, 19]. However, no previous study focused on patients with coexisting hypertension and T2D. In addition, there is a strong association between IR and obesity [20]. However, it is unclear whether obesity-related indicators (BMI and waist circumference, WC) are related to the surrogate indicators of IR. Therefore, in the present study, we aimed to explore the link between the surrogate indicators of IR with the risk of T2D combined with hypertension, as well as the relationship between BMI/WC and TyG to the risk of T2D combined with hypertension, based on two independent cohort studies.

## Methods

### Study design and population

The functional community cohort was established in 2010 in urban Beijing. All participants have received routine annual physical examinations in the physical examination center of Beijing Xuanwu Hospital, Capital Medical University. Most participants (over 80%) are employees of various school companies or local governmental organizations. Both the hospital and university research ethical committees approved the study protocols, and all participants were informed at enrollment. The methods of this study have been described in the previous study [21].

**Fig. 1:**
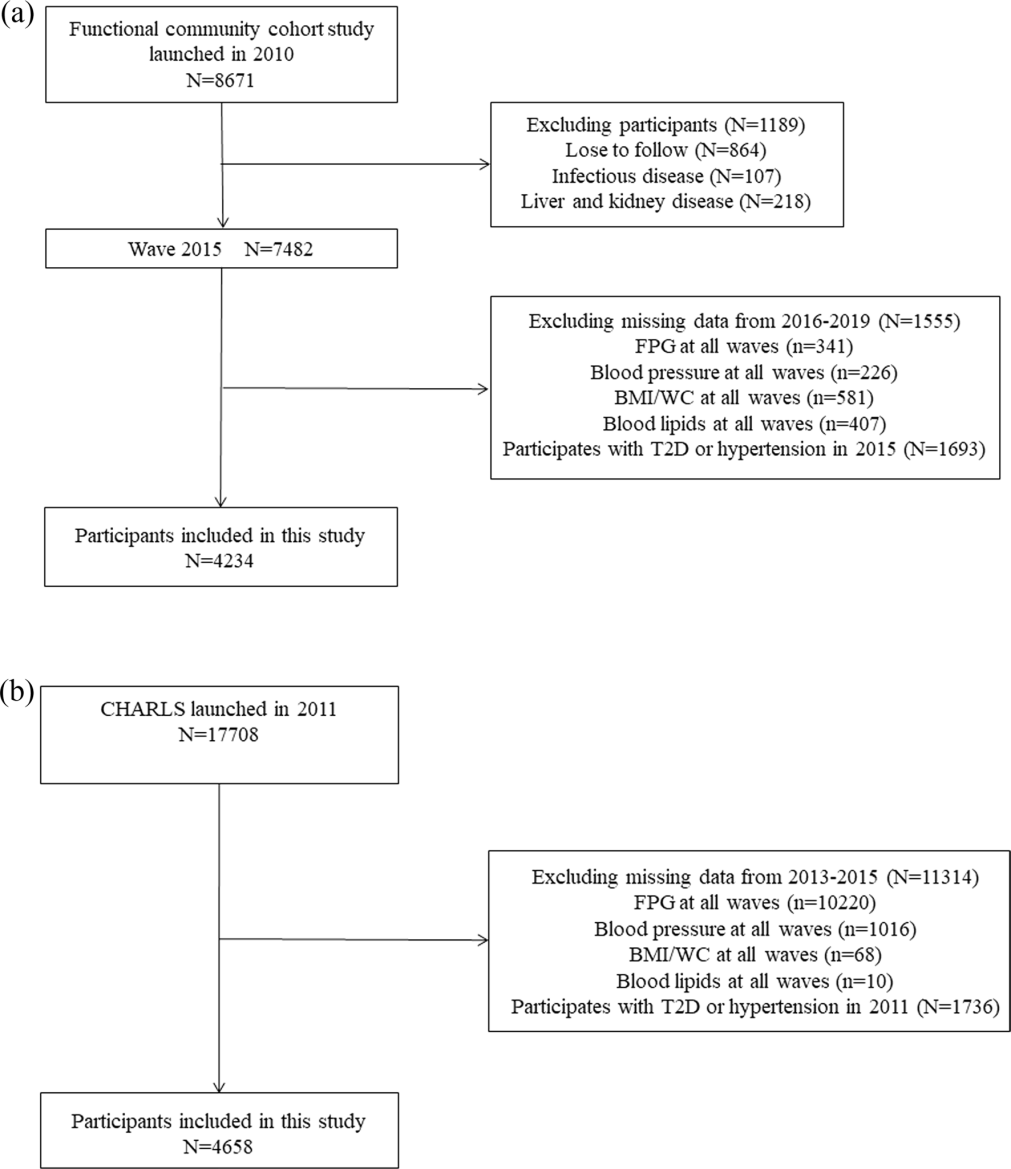
Flow charts illustrating the criteria for participant recruiting and exclusion. FPG, fasting plasma glucose; BMI, body mass index; WC, waist circumference; T2D, type 2 diabetes. **a** The functional community cohort; **b** The China Health and Retirement Longitudinal Study

The present study used survey data from 2015 to 2019. Participants were excluded if they miss simultaneously during follow up from 2016 to 2019 [n = 1555, (FPG: n = 341, blood pressure: n = 226, BMI/WC: n = 581; blood lipids: n = 407)] and diagnosed T2D or hypertension in 2015 (n = 1693), leaving 4234 subjects for our analysis (Fig. 1a).

As most of the participants in the functional community cohort are employees in Beijing, we also analyzed the data extracted from China Health and Retirement Longitudinal Study (CHARLS) to further confirm our conclusions. Briefly, CHARLS is a large-scale longitudinal survey with participants of 45 years of age or older in China. This ongoing cohort study recruited 17,708 participants from 28 provinces in 2011–2012 and follow-up surveys were conducted every 2 years. Details of this study have been described in the previous study [22]. The present study was evaluated based on the cohort from 2011 to 2015. Participants were excluded if they miss simultaneously during follow up from wave 2 (2013) to wave 3 (2015) [n = 11,314, (FPG: n = 10,220, blood pressure: n = 1016, BMI/WC: n = 68; blood lipids: n = 10)] and diagnosed T2D or hypertension in 2011 (n = 1736), leaving 4658 subjects for our analysis (Fig. 1b).

### Measurements

Information of demographic variables including age (continuous), gender (female, male), education levels (primary or less, high school, university or higher), and health-related behaviors including smoking (yes, no), drinking (yes, no), exercise (yes, no) were collected by trained investigators using a structured questionnaire.

Anthropometric tests including weight, height, and waist circumference were measured by trained professionals. BMI was calculated as weight in kilograms divided by height in meters squared. Blood pressure was measured by trained nurses with an automatic blood pressure monitor. Laboratory examinations: Venous blood samples were obtained from the participants in the morning. Biochemical data including triglyceride (TG), total cholesterol (TC), high density lipoprotein cholesterol (HDL), low density lipoprotein (LDL), and fasting plasma glucose (FPG) were estimated by specialized instruments.

We divided the population into three groups based on WC and BMI. BMI ≥ 24 kg/m^2^ were considered overweight [23], WC ≥ 90 cm in males and ≥ 85 cm in female were considered central obesity [24]. Obesity groups include Group1 (not overweight and not centrally obesity), Group 2 (overweight or centrally obese), and group 3 (overweight and centrally obese). Dyslipidemia is defined as TC ≥ 200 mg/dL and/or TG ≥ 200 mg/dL and/or LDL ≥ 130 mg/dL and/or HDL < 40 mg/dL [25].

TyG index was calculated as ln (fasting TG[mg/dL]*fasting glucose[mg/dL]/2). TG/HDL index was calculated as TG [mg/dL]/HDL-C [mg/dL]; METS-IR index was calculated as (ln [(2*FPG) + TG]*BMI)/ (ln [HDL-c]).

### Outcome

T2D was defined as (1) FPG ≥ 7.0 mmol/L; (2) and/or participants told T2D history; (3) and/or participants were currently using antidiabetic medication.

Hypertension was defined as (1) systolic blood pressure (SBP) ≥ 140 mmHg and/or diastolic blood pressure (DBP) ≥ 90 mmHg; (2) and/or participants told hypertension history; (3) and/or participants currently using antihypertension medication.

### Statistical analyses

The data of continuous variables were described by means and standard deviations (SDs). Categorical variables were expressed as proportions. Cox proportional hazards models adjusted for age, gender, education, smoking, drinking, exercise, obesity, and dyslipidemia, were used to evaluate the relationship between the surrogate indicators of IR and T2D/hypertension/T2D combined with hypertension. The adjusted hazard ratios (HR) and 95% confidence intervals (CI) were calculated. The predictive value of BMI, TyG, and Mets-IR index for T2D/hypertension/T2D combined with hypertension were evaluated by the area under curves (AUCs) at 4 years which were calculated by time-dependent receiver operating characteristic (ROC) curves for censored survival data. Next, Cox regression models of the restricted cubic spline with 3 knots of baseline TyG index, adjusted for age, gender, education, smoking, drinking, exercise, obesity, and dyslipidemia, were used to examine the shape of the association between the surrogate indicators of IR with incident T2D/hypertension/T2D combined with hypertension. In addition, subgroup analysis was conducted to evaluate the differential association between TyG index with T2D/hypertension/T2D combined with hypertension among age groups (≤45 or 45–65 or > 65 years), gender (male or female), obesity groups, and dyslipidemia (yes or no).

**Fig. 2:**
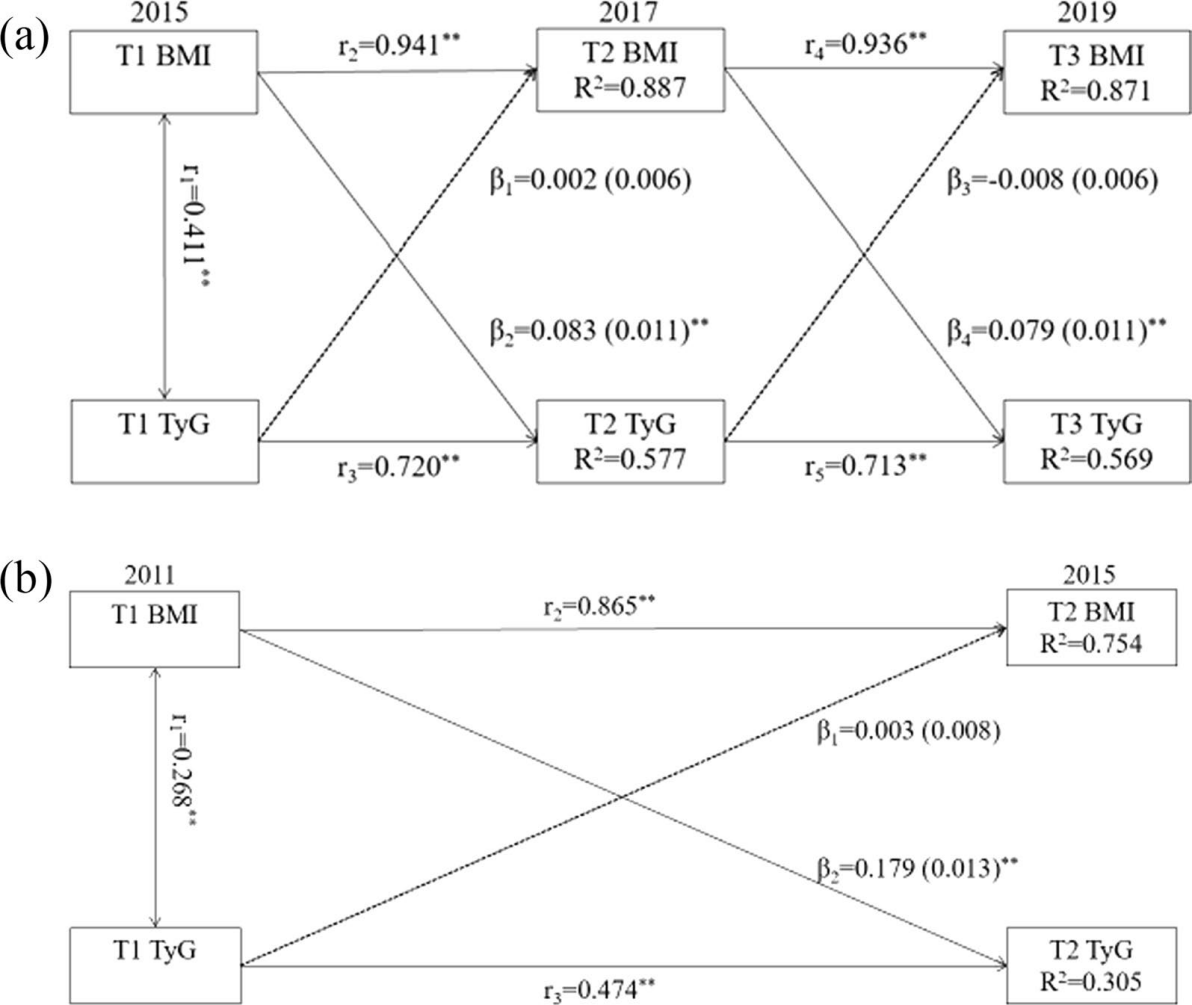
Cross-lagged panel analysis models of BMI and TyG index in two cohort studies, adjusted for age, gender, education, smoking, drinking, and exercise. **a** The functional community cohort, the comparative fitness index = 0.95; **b** The China Health and Retirement Longitudinal Study, the comparative fitness index = 0.95; TyG, triglyceride and glucose index; BMI, body mass index β1, β2, β3 and β4 indicate cross-lagged path coefficients; r1, indicates synchronous correlation; r2, r3, r4 and r5 indicates tracking correlations; R^2^ indicates variance explained; ^*^P < 0.05, ^**^P < 0.001

The cross-lagged panel analysis was applied to analyze the association between baseline BMI/WC and follow-up TyG index and the impact of baseline TyG index on follow-up BMI/WC with adjustment for age, gender, education, smoking, drinking, and exercise. The previous study has introduced the application of cross-lagged panel analysis [26], which is a statistical method to deal with the bidirectional relationship between two (or more) observed variables changing with time. In this present study, the cross-lagged panel analysis was conducted using variables measured at three time points in the functional cohort study and two time points in the CHARLS. Figure 2a illustrated the theoretical models, the path coefficients β1 and β3 describe the relationship between baseline TyG index and follow-up BMI, while the path coefficients β2 and β4 describe the relationship between baseline BMI and follow-up TyG index. Both BMI and TyG index values were standardized with Z-transformation (mean = 0; SD = 1). It shows that the standardized regression coefficient of BMI was greater than that of the TyG index. Besides, the comparative fitness index (CFI) was applied to assess the model fit, CFI > 0.90 indicating a relatively good fit.

When the temporal relationships between BMI (WC) and TyG index had been established in the cross-lagged panel analysis, the mediation model would be fitted to explore whether the association of BMI (WC) with T2D/hypertension/T2D combined with hypertension was mediated by TyG index after adjusting for age, gender, education, smoking, drinking, and exercise. In the present mediation models, we used baseline BMI (WC) as the predictor variable, follow-up TyG index as the mediator variable, and T2D/hypertension/T2D combined with hypertension as the outcome.

The cross-lagged panel analysis and mediation analysis were performed by Mplus software version 7.0. Other analyses were performed using the SPSS software version 24.0, and R software version 4.0.3. All statistical tests were 2-sided, and statistical significance was set at *P* < 0.05.

## Results

### Baseline characteristics of the study participants

A total of 4234 participants from the functional community cohort [mean (SD) age, 49.42 (13.61) years; 45.5% males; median follow-up duration, 4.00 (IQR, 3.97–4.03) years], and 4658 participants from CHARLS [mean (SD) age, 58.01 (9.05) years; 46.3% males; median follow-up duration, 4.00 (IQR, 3.92-4.00) years] were included in our final analysis. In the functional community cohort, during the 4-year follow-up period, a total of 277 participants had developed T2D, 1309 participants had developed hypertension and 141 participants had developed T2D combined with hypertension. In the CHARLS, 506 participants had developed T2D, 1458 participants had developed hypertension and 203 participants had developed T2D combined with hypertension. Table 1 presents mean levels of study variables among participants at baseline.

**Table 1:** Characteristics regarding the study variables at baseline in two cohort studies

Characteristic	Cohort 1 (N = 4234)	Cohort 2 (N = 4658)
Age (years)	49.42 (13.61)	58.01 (9.05)
Gender (Male, N, %)	1928 (45.5)	2156 (46.3)
Education level		
Primary or less (N, %)	90 (2.1)	3283 (70.5)
High school (N, %)	1125 (26.6)	1324 (28.4)
University or higher (N, %)	3019 (71.3)	50 (1.1)
Smoking (Yes, N, %)	264 (6.2)	1459 (31.3)
Drinking (Yes, N, %)	425 (10.0)	1606 (34.5)
Exercise (Yes, N, %)	3047 (72.0)	1808 (38.8)
BMI (kg/m^2^)	24.28 (3.23)	22.97 (3.31)
Waistline (cm)	80.43 (9.66)	83.87 (9.30)
DBP (mmHg)	73.16 (8.51)	73.62 (11.22)
SBP (mmHg)	118.09 (12.30)	125.14 (18.57)
FPG (mg/dl)	92.78 (10.04)	105.88 (27.47)
TG (mg/dl)	122.88 (84.77)	126.81 (100.52)
HDL (mg/dl)	67.49 (16.49)	52.11 (15.44)
LDL (mg/dl)	117.14 (31.75)	114.96 (33.79)
TC (mg/dl)	183.00 (33.66)	191.72 (37.84)
TyG	8.48 (0.57)	8.62 (0.63)
TG/HDL	2.21 (6.81)	3.10 (5.71)
Mets-IR	33.45 (6.61)	34.40 (7.38)
Obesity Group		
Group 1	2049 (48.4)	2614 (56.1)
Group 2	1262 (29.8)	793 (17.0)
Group 3	923 (21.8)	1251 (26.9)
Dyslipidemia (Yes, N, %)	2076 (49.0)	2669 (57.3)

Cohort 1, The functional community cohort; Cohort 2, The China Health and Retirement Longitudinal Study; BMI, body mass index; DBP, diastolic blood pressure; SBP, systolic blood pressure; FPG, fasting plasma glucose; TG, triglyceride; TC, high cholesterol; LDL-C, low-density lipoprotein cholesterol; HDL, high-density lipoprotein cholesterol

### Association between the surrogate indicators of IR with T2D and hypertension

**Fig. 3:**
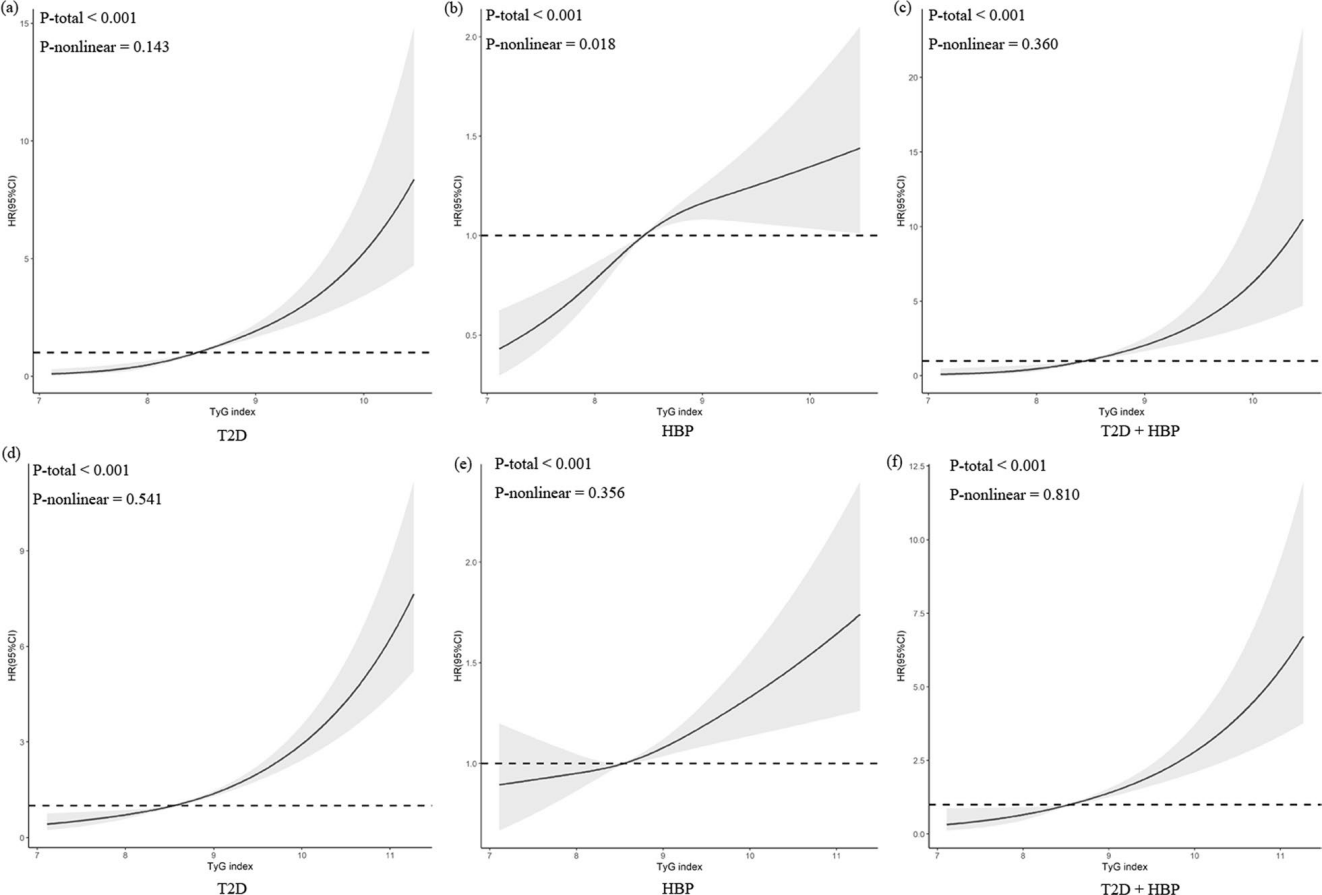
The associations of TyG index with risk of T2D and hypertension. Data were fitted using Cox regression models of the restricted cubic spline with 3 knots of baseline TyG index, adjusted for age, gender, education, smoking, drinking, exercise, obesity and dyslipidemia. **a–c** The functional community cohort; **d**–**f** The China Health and Retirement Longitudinal Study. HBP, hypertension; T2D, type 2 diabetes; TyG, triglyceride-glucose index, HR hazard ratio, CI confidence interval

Table 2 presents the results from the multivariate Cox proportional hazards models. The results based on the functional community cohort showed that higher TyG and Mets-IR were related to higher risks of developing T2D (TyG: adjusted HR = 3.16, 95% CI 2.47–4.04; Mets-IR: adjusted HR = 1.04, 95%CI: 1.03–1.05), hypertension (TyG: adjusted HR = 1.39, 95% CI: 1.23–1.57; Mets-IR: adjusted HR = 1.03, 95%CI: 1.02–1.04), and T2D combined with hypertension (TyG: adjusted HR = 3.46, 95%CI: 2.43–4.93; Mets-IR: adjusted HR = 1.04, 95%CI: 1.03–1.06). However, the association between TG/HDL with T2D (hypertension) was not statistically significant in the multivariate models. Results were consistent in the link of TyG index, Mets-IR with T2D/hypertension/T2D combined with hypertension in CHARLS. While the link of TG/HDL with T2D/hypertension/T2D combined with hypertension was statistically significant after adjusting for covariates in CHARLS. In the restricted cubic spline regression models in two cohort studies, the relationship between the TyG index and risk of incident T2D combined with hypertension was linear (*P*_Nonlinear_ > 0.05) (Fig. 3c, f).

**Table 2:** Prospective associations between baseline surrogate indexes of IR with follow-up incident T2D and HBP in two cohort studies

	T2D		HBP		T2D + HBP	
	HR (95%CI)	*P*	HR (95%CI)	*P*	HR (95%CI)	*P*
Cohort 1						
TyG	3.16 (2.47,4.04)	< 0.001	1.39 (1.23,1.57)	< 0.001	3.46 (2.43,4.93)	< 0.001
TG/HDL	1.00 (1.00,1.01)	0.361	1.00 (1.00,1.01)	0.054	1.00 (0.99,1.02)	0.514
Mets-IR	1.04 (1.03,1.05)	< 0.001	1.03 (1.02,1.04)	< 0.001	1.04 (1.03,1.06)	< 0.001
Cohort 2						
TyG	2.05 (1.81,2.32)	< 0.001	1.17 (1.07,1.28)	< 0.001	2.02 (1.67,2.44)	< 0.001
TG/HDL	1.01 (1.00,1.02)	0.003	1.01 (1.00,1.02)	0.006	1.01 (1.00,1.02)	0.035
Mets-IR	1.05 (1.04,1.06)	< 0.001	1.01 (1.00,1.02)	0.013	1.05 (1.03,1.07)	< 0.001

Cohort 1, The functional community cohort; Cohort 2, The China Health and Retirement Longitudinal Study; HR, hazard ratio; CI, confidence intervals; HBP, hypertension; T2D, type 2 diabetes; TyG, triglyceride and glucose index, TG/HDL, triglyceride to high-density lipoprotein ratio, Mets-IR, metabolic score for IR.

Cox proportional hazards models adjusted for age, gender, education, smoking, drinking, exercise, obesity and dyslipidemia

### ROCs of BMI, TyG index, and Mets-IR for T2D and hypertension

**Fig. 4:**
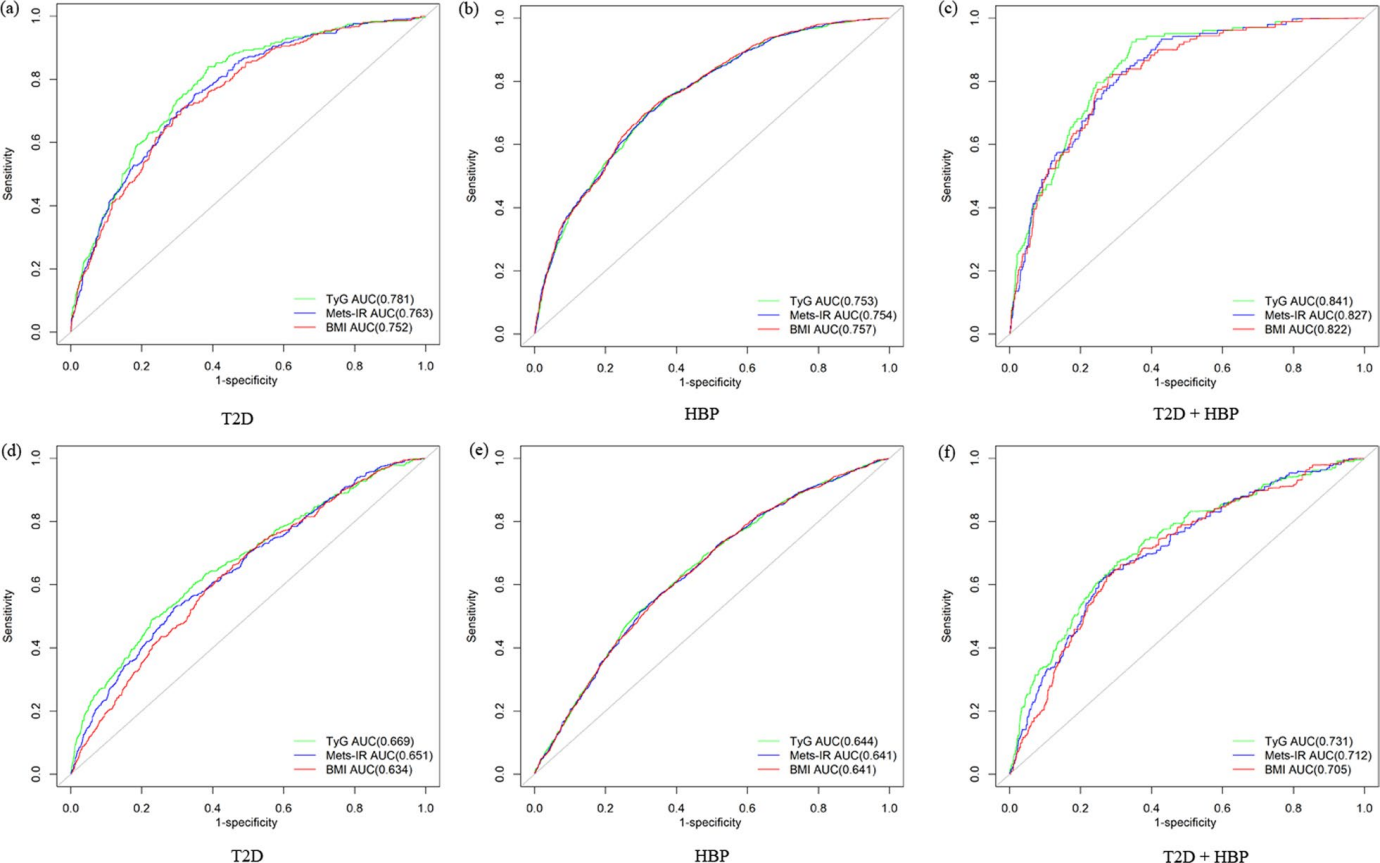
Time-dependent receiver operating characteristic (ROC) curves of TyG, Mets-IR and BMI for T2D and HBP at 4 years in two cohort studies. **a–c** The functional community cohort; **d**–**f** The China Health and Retirement Longitudinal Study. HBP, hypertension; T2D, type 2 diabetes; TyG, triglyceride and glucose index, Mets-IR, metabolic score for IR; BMI, body mass index

Time-dependent ROC analysis revealed the AUC at 4 years. In the functional community cohort, the highest AUC was demonstrated by TyG (AUC = 0.781, cut-off values: 8.70), followed by Mets-IR (AUC = 0.763, cut-off values: 34.73), and BMI (AUC = 0.752, cut-off values: 24.21) for T2D. The AUC was closely demonstrated by TyG (AUC = 0.753, cut-off values: 9.34), Mets-IR (AUC = 0.754, cut-off values: 34.16), and BMI (AUC = 0.757, cut-off values: 24.38) for hypertension. The highest AUC was demonstrated by TyG (AUC = 0.841, cut-off values: 7.83), followed by Mets-IR (AUC = 0.827, cut-off values: 36.48), and BMI (AUC = 0.822, cut-off values: 26.48) for T2D combined with hypertension (Fig. 4a–c and Additional file 1: Table S1). Although the diagnostic performance decreases in CHARLS, it is still statistically significant (Fig. 4d–f and Additional file 1: Table S1).

### Subgroup analyses

**Fig. 5:**
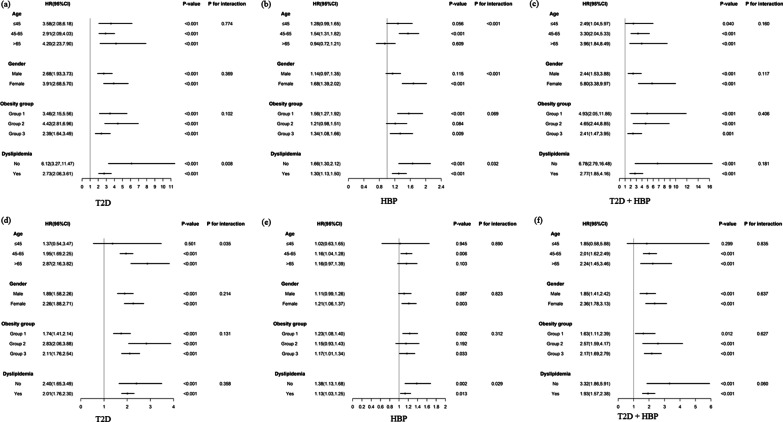
Cox regression models of TyG index for follow-up incident T2D and HBP in subgroups stratified by gender, age, obesity and dyslipidemia in two cohort studies. Adjusted for education, smoking, drinking, exercise, and age/ gender/ obesity/ dyslipidemia. **a**–**c** The functional community cohort; **d**–**f** The China Health and Retirement Longitudinal Study. HR, hazard ratio; CI, confidence intervals; HBP, hypertension; T2D, type 2 diabetes; TyG, triglyceride and glucose index

We performed subgroup analyses to stratify the link of TyG index with follow-up T2D/hypertension/T2D combined with hypertension by age, gender, obesity, and dyslipidemia, as provided in Fig. 5. These subgroup variables may not influence our results for T2D combined with hypertension, because the prospective associations of baseline TyG index with incident T2D combined with hypertension between both stratifications showed similar associations in two cohort studies. No interaction was found between subgroup variables and the association of the TyG index with the risk of T2D combined with hypertension in two cohorts (Fig. 5c, f). While results of interaction between subgroup variables and the association of the TyG index with the risk of T2D or hypertension were different in the two cohorts.

### Bidirectional relationship between BMI/WC and TyG index

Figure 2 presents the results of the bidirectional relationship between BMI and TyG index examined by the cross-lagged panel analysis in two cohorts. In the functional community cohort, β_baseline BMI→follow−up TyG_ (β2 = 0.083, β4 = 0.079) were statistically significant (*P* < 0.001). But β_baseline TyG to follow−up BMI_ (β1 = 0.002, β3 =-0.008) were not statistically significant (Fig. 2a). It indicated that changes in BMI might precede the TyG index. The results of the cross-lagged panel analysis in CHARLS were consistence with that of the functional community cohort (Fig. 2b). And the association of WC and TyG was consistence with those of BMI and TyG index (Additional file 1: Figure S3).

### The mediation effect of TyG index on BMI/WC to T2D and hypertension

**Fig. 6:**
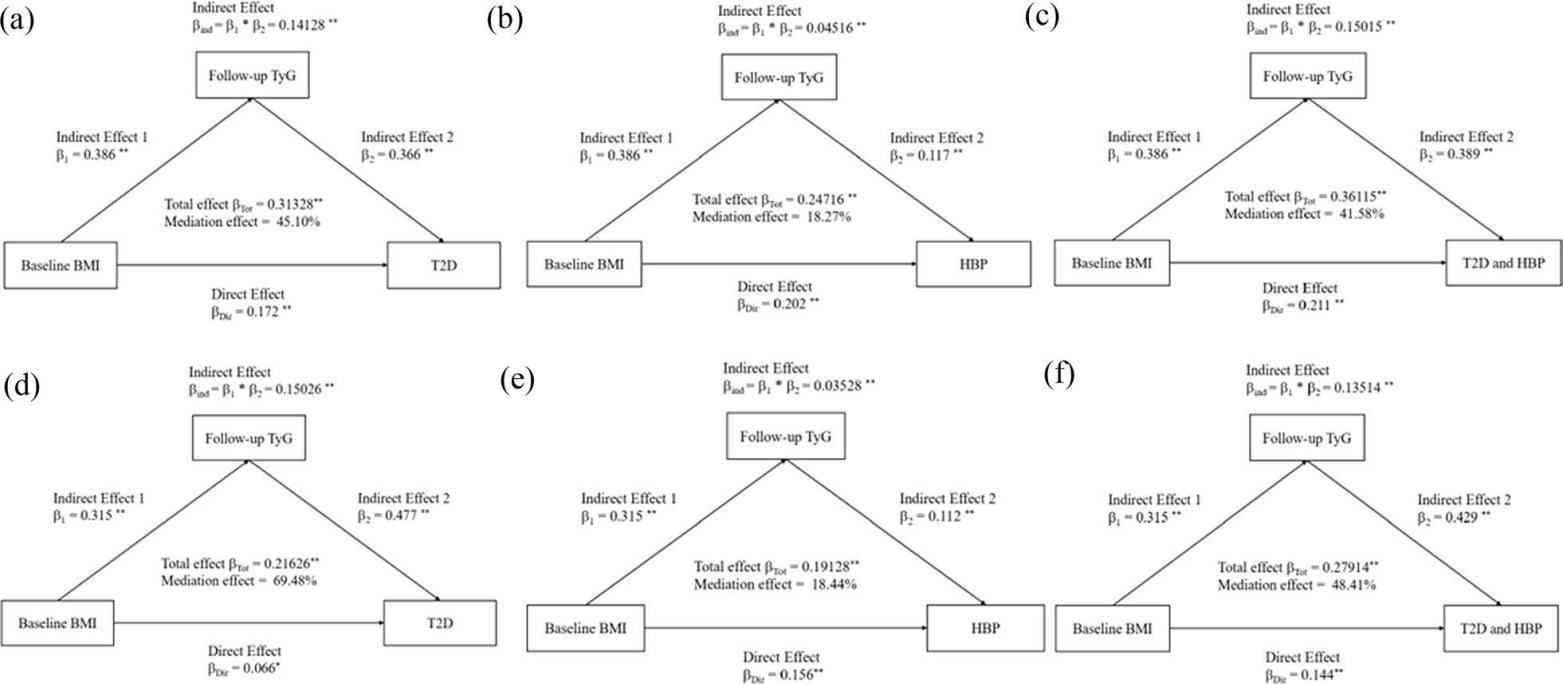
Mediation effect of TyG on BMI–T2D and hypertension, adjusted for age, gender, education, smoking, drinking, and exercise. **a–c** The functional community cohort; **d–f** The China Health and Retirement Longitudinal Study. HBP, hypertension; T2D, type 2 diabetes; TyG, triglyceride and glucose index; BMI, body mass index; ^*^P < 0.05, ^**^P < 0.001. Overall Indirect Effect (β_ind_) = Indirect Effect 1 (β_1_) * Indirect Effect 2 (β_2_). Total Effect (β_Tot_) = Overall Indirect Effect (β_ind_) + Direct Effect (β_Dir_). Mediation Effect (%) = Overall Indirect Effect (β_ind_)/ Total Effect (β_Tot_) × 100%

Figure 6 presents the mediation effects of the follow-up TyG index on the association between baseline BMI and follow-up risk of T2D/hypertension/T2D combined with hypertension. In the functional community cohort, the total effect of baseline BMI on the follow-up T2D/hypertension/T2D combined with hypertension was 0.31328, 0.24716, and 0.36115, respectively. The β1 and β2 were used to calculate the overall indirect effect for the TyG index (βind = 0.14128 for T2D, βind = 0.04516 for HBP, βind = 0.15015 for T2D combined with HBP; both *P* < 0.001). The percentages of the total effect mediated by the TyG index were estimated at 45.10% for T2D, 18.27% for HBP, and 41.58% for T2D combined with HBP (Fig. 6a–c). The results of the mediation analysis in CHARLS were consistence with those of the functional community cohort (Fig. 6d, e). When WC was applied as a predictor variable, the results were consistent with those of BMI (Additional file 1: Figure S4).

## Discussion

Based on two cohort studies among Chinese adults, we found positive associations between the surrogate indicators of IR (TyG index and Mets-IR) with the risk of T2D/hypertension/T2D combined with hypertension. TyG index shows a good predictive ability for T2D combined with hypertension. The association between TyG index with T2D combined with hypertension is not affected by age, gender, obesity, and dyslipidemia. Besides, the TyG index partially mediated the effects of BMI (WC) on the follow-up risk of T2D/hypertension/T2D combined with hypertension.

It is well-recognized that IR raises the risk of T2D, hypertension, dyslipidemia, and cardiovascular disease [27]. We found higher TyG index was related to higher risks of T2D and hypertension, which is consistent with existing evidence. Several cross-sectional studies showed that the TyG index could serve as a surrogate indicator of IR with T2D and hypertension [28, 29]. Findings from the Korean Genome and Epidemiology Study showed that the ROC of the TyG index was 0.784, which showed better predictability for the prevalence of T2D than HOMA-IR (ROC: 0.728) [30]. The results of several cohort studies have also confirmed the relationship between the TyG index with incident T2D and hypertension [30–32]. Results of a 12-year cohort study conducted in Korea showed TyG index is a predictor of incident T2D. Besides, the TyG index and hypertension have a well-established high association in large longitudinal population studies [16, 33]. Our study also confirmed that Mets-IR, newly proposed surrogate indicators for IR, is associated with the risk of developing T2D and hypertension. Several studies support our conclusions [34, 35]. However, the results of the link between TG/HDL with T2D and hypertension were different in the two cohort studies. Evidence from cohort studies confirmed that TG/HDL ratio was positively associated with the risk of T2D and hypertension [36–38]. The reason for the different results may be the variability of the study population. In addition, TG/HDL may not be accurate to assess IR by lipid markers alone. Few studies have focused on the association between the surrogate indicators of IR and the risk of developing T2D combined with hypertension. Our study found that the TyG index has high predictive power for T2D, hypertension, and T2D combined with hypertension. The TyG index can be used widely in large-scale observational cohorts to find those who are at higher risk of T2D and hypertension.

According to several studies, there is a connection between IR and obesity [13, 39]. In this study, we examined the temporal relationship between obesity and IR using cross-lagged path analysis. The results revealed that the paths from BMI (WC) to TyG were more significant than those from TyG to BMI (WC), indicating that BMI change may precede TyG index change. This important path from BMI to TyG is consistent with the previous study [33]. Dysfunctional obese adipose tissue plays a vital role in the emergence of IR [40]. Pro-inflammatory cytokines are released from tissue macrophages and directly reduce insulin sensitivity, which may contribute to the development of IR associated with obesity [41, 42]. The results of the mediation analysis indicated that IR partially mediated the association between obesity and the risk of T2D and hypertension. Obesity causes the development of IR, which then leads to T2D and hypertension.

This study had several advantages. The risk of developing incident T2D/hypertension/T2D combined with hypertension was evaluated in our study using simple surrogate indicators of IR among Chinese adults in two cohort studies. We highlighted the relationship between the TyG index and the incidence of T2D combined with hypertension for the first time. We also used cross-lagged panel analysis and mediation analysis to explore the relationship between BMI/WC and TyG to the risk of T2D/hypertension/T2D combined with hypertension. In both cohort studies, our results were consistent. Therefore, our results can be considered persuasive. However, our study is susceptible to some restrictions. First, we could not obtain information on dietary habits and medication information, which may affect our results. Second, as an observational study, we cannot rule out the possibility of residual or unmeasured confounders, although adjustments have been made for some potential confounders. Third, we were unable to obtain the insulin levels of participants to compare the association between the surrogate indicators of IR and traditional indicators. Furthermore, the participants in the two cohort studies were limited to the Chinese population, so the results should be interpreted with caution. Due to these limitations, further studies should be conducted to clarify the above factors.

## Conclusion

In conclusion, the TyG index was significantly associated with the risk of T2D and hypertension in Chinese adults. TyG index could be a valuable marker for predicting the incidence of T2D and hypertension. Besides, BMI (WC) change may precede TyG change, TyG index partially mediated the effect of BMI (WC) on follow-up risk of T2D and hypertension. TyG index could be used widely in large-scale observational cohorts to identify individuals at high risk for T2D and hypertension.

## Supplementary Information

**Additional file 1.** Supplementary material.

## Data Availability

The datasets are available from the corresponding author upon reasonable request.

## Abbreviations

T2D: Type 2 diabetes
IR: Insulin resistance
EICT: Euglycemic insulin clamp technique
TyG: Triglyceride and glucose index
TG/HDL: Triglyceride to high-density lipoprotein ratio
Mets-IR: Metabolic score for IR
BMI: Body mass index
WC: Waist circumference
TG: Triglyceride
TC: Total cholesterol
HDL: High density lipoprotein cholesterol
LDL: Low density lipoprotein
FPG: Fasting plasma glucose
SBP: Systolic blood pressure
DBP: Diastolic blood pressure
HR: Hazard ratio
CI: Confidence interval
AUC: Area under curve
ROC: Receiver operating characteristic
CFI: Comparative fitness index
