# Supplementary material for: Association between surrogate indicators of insulin resistance and risk of type 2 diabetes combined with hypertension among Chinese adults: two independent cohort studies
Source: Nutr Metab (Lond). 2022 Dec 29;19:85. doi: 10.1186/s12986-022-00720-1 (PMC9798583; doi:10.1186/s12986-022-00720-1)
Supplement: Supplementary file 1 — Additional file 1. Supplementary material. [file 12986_2022_720_MOESM1_ESM.docx]

Table S1. The area under the curve and optimal cut-off values in two cohorts

| Outcome | Indicators | AUC | Sensitivity (%) | Specificity (%) | Cut-off value |
| --- | --- | --- | --- | --- | --- |
| Cohort 1 |  |  |  |  |  |
| T2D | TyG | 0.781 | 84.0 | 61.4 | 8.70 |
|  | Mets-IR | 0.763 | 75.1 | 65.3 | 34.73 |
|  | BMI | 0.752 | 84.0 | 67.9 | 24.21 |
| HBP | TyG | 0.753 | 69.0 | 69.1 | 9.34 |
|  | Mets-IR | 0.754 | 70.2 | 67.8 | 34.16 |
|  | BMI | 0.757 | 69.6 | 69.1 | 24.38 |
| T2D+HBP | TyG | 0.841 | 92.5 | 65.7 | 7.83 |
|  | Mets-IR | 0.827 | 83.0 | 68.4 | 36.48 |
|  | BMI | 0.822 | 81.3 | 72.3 | 26.48 |
| Cohort 2 |  |  |  |  |  |
| T2D | TyG | 0.669 | 48.9 | 77.2 | 8.96 |
|  | Mets-IR | 0.651 | 52.7 | 70.9 | 33.77 |
|  | BMI | 0.634 | 59.0 | 61.7 | 29.56 |
| HBP | TyG | 0.644 | 51.3 | 71.0 | 8.62 |
|  | Mets-IR | 0.641 | 54.0 | 67.8 | 36.02 |
|  | BMI | 0.641 | 57.2 | 64.2 | 21.20 |
| T2D+HBP | TyG | 0.731 | 67.2 | 69.7 | 8.80 |
|  | Mets-IR | 0.712 | 62.8 | 72.9 | 33.88 |
|  | BMI | 0.705 | 62.7 | 72.6 | 24.20 |

Cohort 1, The functional community cohort; Cohort 2, The China Health and Retirement Longitudinal Study. HBP, hypertension; T2D, type 2 diabetes; TyG, triglyceride and glucose index, Mets-IR, metabolic score for IR; BMI, body mass index.


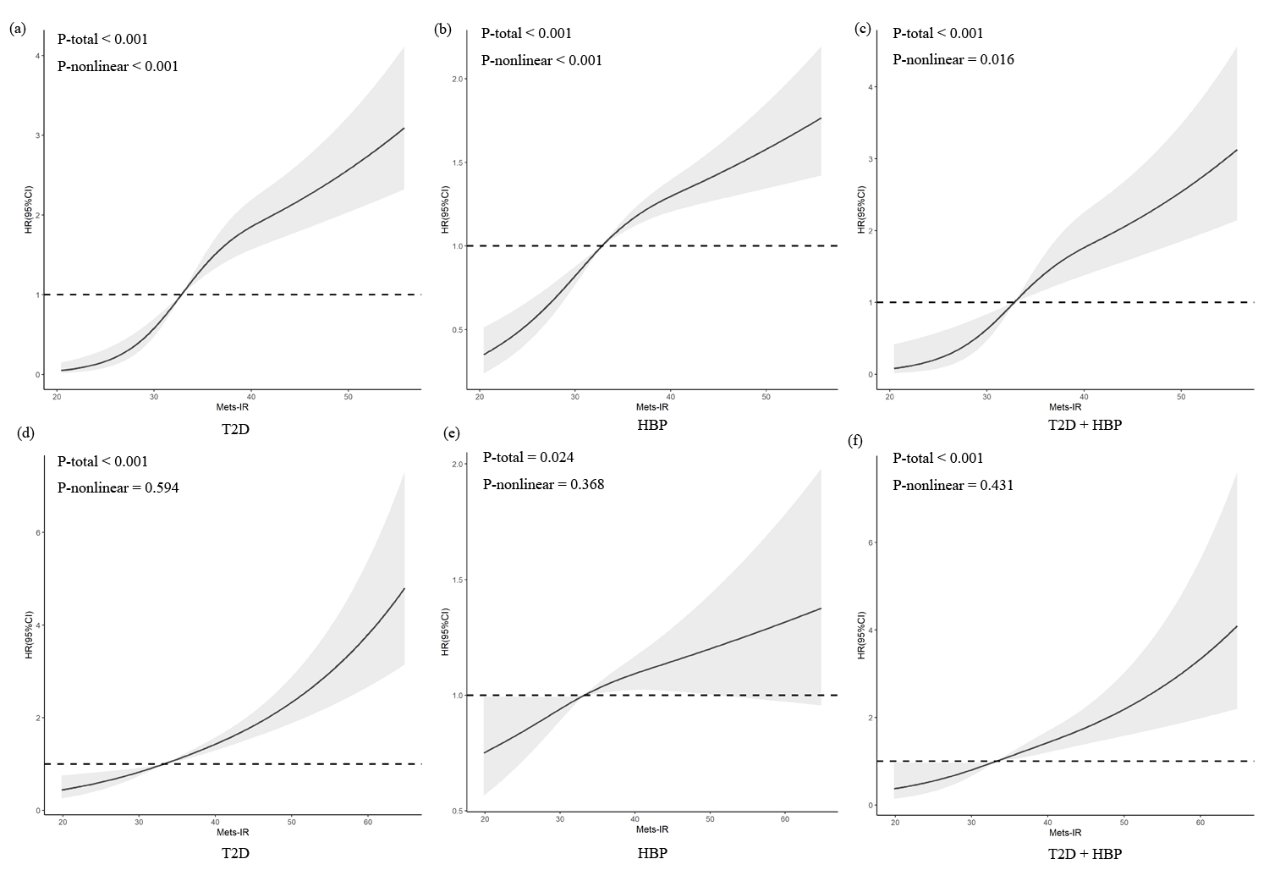


Figure S1. The associations of Mets-IR with risk of T2D and hypertension. Data were fitted using Cox regression models of the restricted cubic spline with 3 knots of baseline Mets-IR, adjusted for age, gender, education, smoking, drinking, exercise, obesity and dyslipidemia. (a-c): The functional community cohort; (d-f): The China Health and Retirement Longitudinal Study.

HBP, hypertension; T2D, type 2 diabetes; Mets-IR, metabolic score for IR, HR hazard ratio, CI confidence interval.


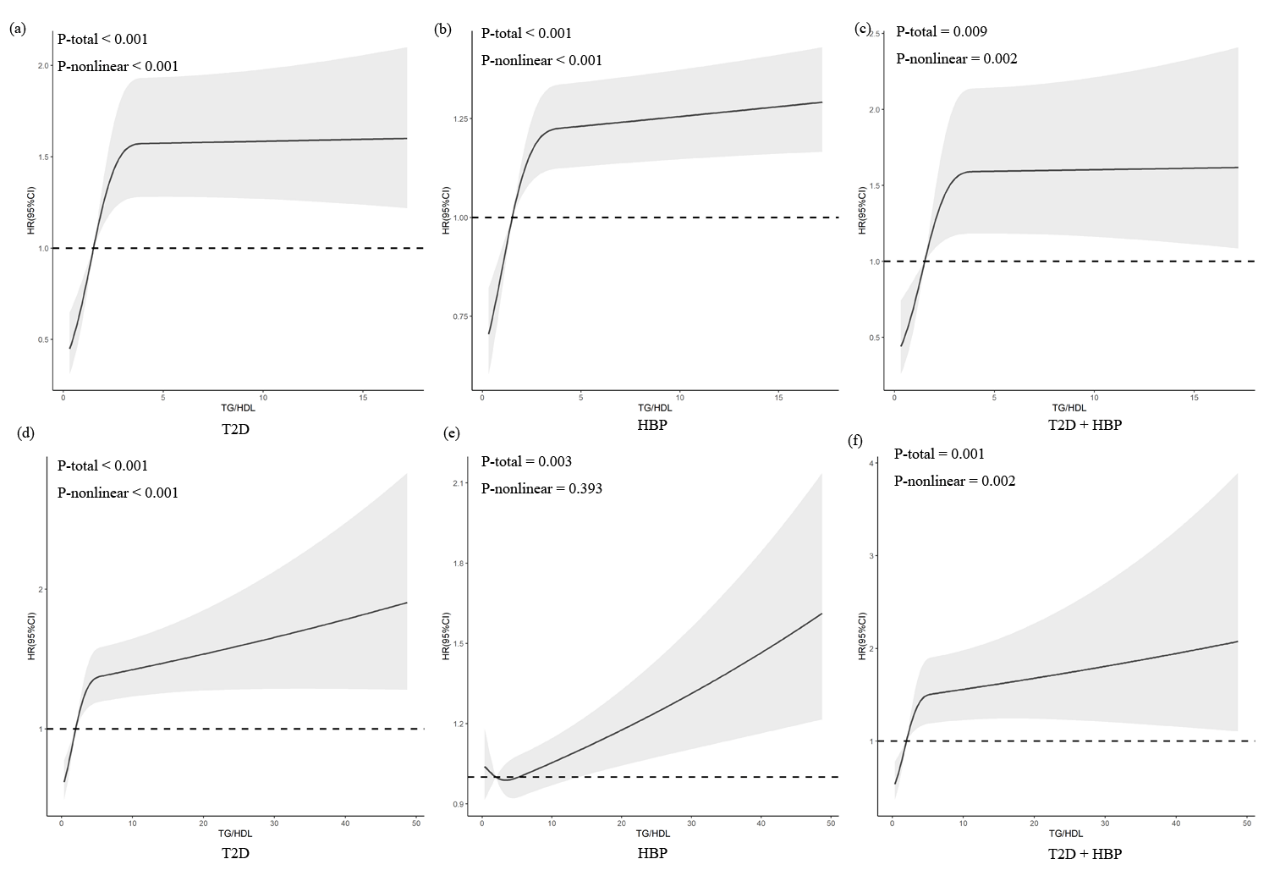


Figure S2. The associations of TG/HDL with risk of T2D and hypertension. Data were fitted using Cox regression models of the restricted cubic spline with 3 knots of baseline Mets-IR, adjusted for age, gender, education, smoking, drinking, exercise, obesity and dyslipidemia. (a-c): The functional community cohort; (d-f): The China Health and Retirement Longitudinal Study.

HBP, hypertension; T2D, type 2 diabetes; TG/HDL, triglyceride to high-density lipoprotein ratio, HR hazard ratio, CI confidence interval.


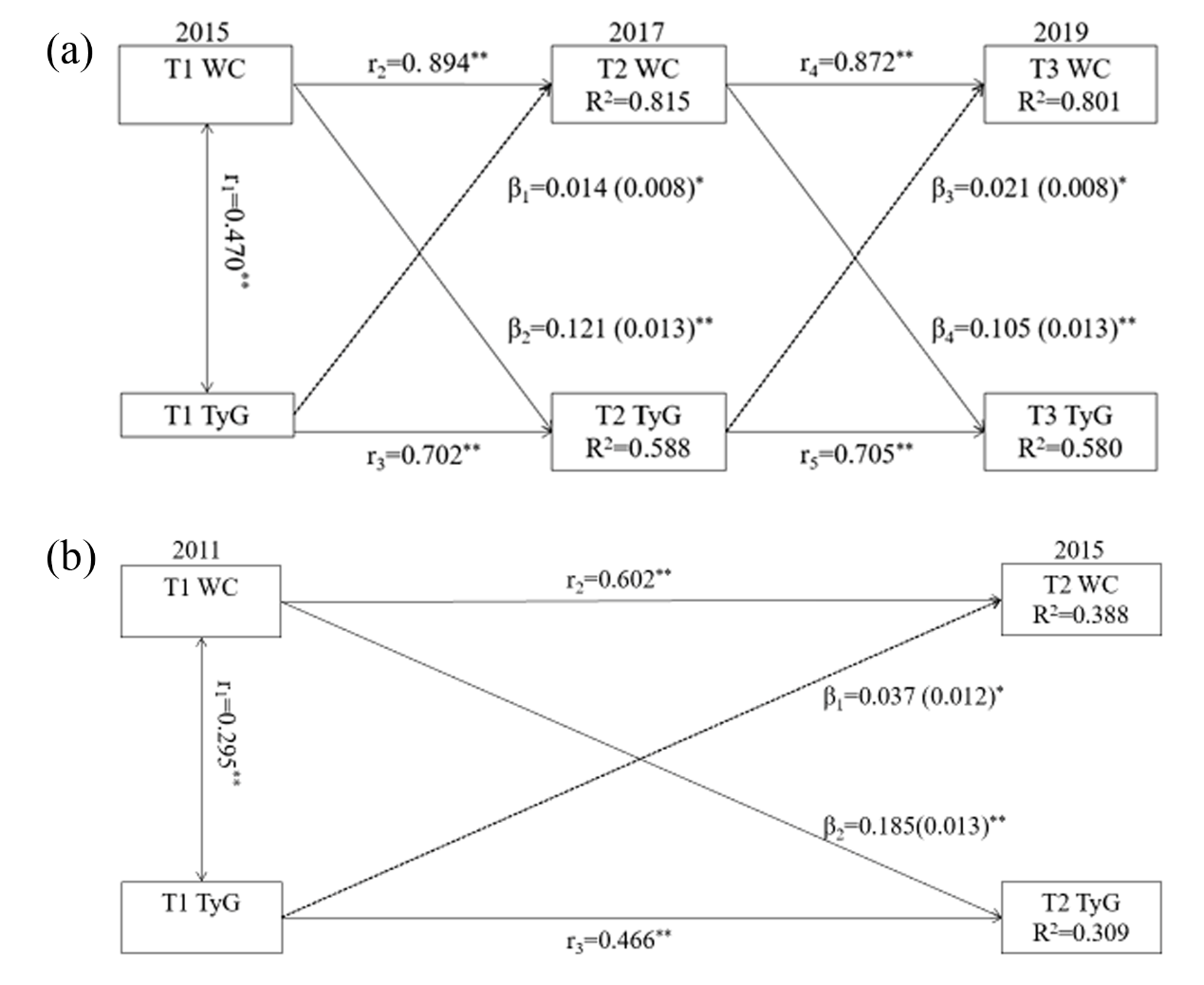


Figure S3. Cross-lagged panel analysis models of WC and TyG index in two cohort studies, adjusted for age, gender, education, smoking, drinking, and exercise. (a): The functional community cohort, the comparative fitness index=0.86; (b): The China Health and Retirement Longitudinal Study, the comparative fitness index=0.98; TyG, triglyceride and glucose index; WC, waist circumference.

β1, β2, β3 and β4 indicate cross-lagged path coefficients; r1, indicates synchronous correlation; r2, r3, r4 and r5 indicates tracking correlations; R^2^ indicates variance explained; ^*^*P*<0.05, ^**^*P*<0.001.


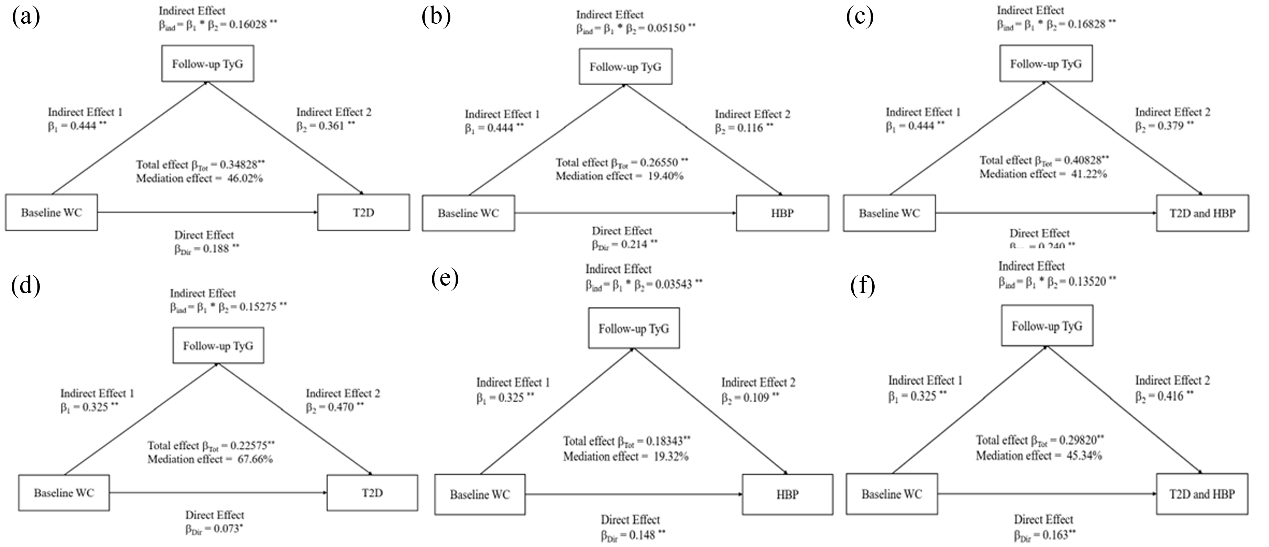


Figure S4. Mediation effect of TyG on WC–T2D and hypertension, adjusted for age, gender, education, smoking, drinking, and exercise. (a-c): The functional community cohort; (d-f): The China Health and Retirement Longitudinal Study. HBP, hypertension; T2D, type 2 diabetes; TyG, triglyceride and glucose index; WC, waist circumference; ^*^*P*<0.05, ^**^*P*<0.001.

Overall Indirect Effect (β_ind_) = Indirect Effect 1 (β_1_) * Indirect Effect 2 (β_2_)

Total Effect (β_Tot_) = Overall Indirect Effect (β_ind_) + Direct Effect (β_Dir_)

Mediation Effect (%) = Overall Indirect Effect (β_ind_)/ Total Effect (β_Tot_) × 100%
